# The Separation of Chlorobenzene Compounds from Environmental Water Using a Magnetic Molecularly Imprinted Chitosan Membrane

**DOI:** 10.3390/polym14153221

**Published:** 2022-08-08

**Authors:** Guizhen Li, Jinyao Wang, Peng Zhu, Ying Han, Anqi Yu, Junhong Li, Zhaomei Sun, Kyung Ho Row

**Affiliations:** 1 School of Chemistry and Chemical Engineering, Linyi University, Linyi 276005, China; 2 Department of Chemistry and Chemical Engineering, Inha University, Incheon 402751, Korea

**Keywords:** magnetic molecularly imprinted chitosan membrane, membrane separation, chlorobenzene compounds, environmental water

## Abstract

In this work, a magnetic molecularly imprinted chitosan membrane (MMICM) was synthesized for the extraction of chlorobenzene compounds in environmental water using the membrane separation method. The optimal extraction amount for chlorobenzene (9.64 mg·L^−1^) was found to be a 1:2 solid to liquid ratio, with a 20 min extraction time and 35 °C extraction temperature. This method proved to be successfully applied for the separation and trace quantification of chlorobenzene compounds in environmental water, with the limit of detection (LOD) (0.0016–0.057 ng·L^−1^), limit of quantification (LOQ) (0.0026–0.098 ng·L^−1^), and the recoveries ranging (89.02–106.97%).

## 1. Introduction

Chlorobenzenes are a kind of organic chemical raw material and are mainly used as intermediates in dyestuffs, medicines, pesticides and in organic synthesis, as they carry the disadvantages of not being easy to degrade and of being harmful to the environment [1,2]. There is a great need to develop low-cost, easy-operation, high-efficiency, environmentally protective techniques for the separation of trace chlorobenzenes compounds in water samples, due to their toxicity, environmental damage potential, and the fact that they pose a great threat to human health [3]. Until now, many pretreatment methods have been developed for the separation of chlorobenzenes from environmental water, including solid-phase extraction (SPE), solid-phase microextraction (SPME), liquid–liquid extraction (LLE), and single-drop microextraction (SDME) [4,5]. However, given the trace concentrations in environmental matrices, it is still difficult to conveniently and precisely detect chlorobenzenes in real environmental matrices using these methods [6].

Membrane technology has been widely applied in the purification and separation fields, including gas separation, sewage treatment, the leather industry, biodiesel purification, the food industry and the fermentation industry [7,8,9]. This technology not only solves many technical problems, but also provides numerous and better advantages with respect to environmental protection, production improvement, and energy conservation [10]. Consequently, membrane separation has received increasing attention in the field of separation, owing to its advantages of lower energy, shorter extraction time, high separation efficiency and greater flexibility, and, in this way, it has become a more attractive technology for large-scale production in industry [11,12,13]. The research results have shown that the materials used for membrane separation can be organic, inorganic or composite materials, and the selection of effective materials is very important for the separation process, including the acquisition of good separation results, high separation efficiency and large-scale applications [14,15]. 

Molecularly imprinted polymers (MIPs) are a kind of recognition and separation material, and they create recognition sites for one or for a class of molecules by combining functional monomers, template molecules, initiators, cross-linking agents, etc. [16]. MIPs have been widely used in various fields because of their specificity, designability, high selectivity and stability [17]. Bulk polymerization is a traditional method for the synthesis of MIPs, and through polymerization, the template molecules are eluted off, and free recognition sites for the analytes are created [18]. At present, MIPs are being rapidly developed and widely used in extraction and separation, in chromatographic separation and in chemical sensors [19,20]. 

Chitosan is considered as a functional biomaterial with greater application potential than cellulose, and it is the product of natural polysaccharide chitin with the removal of some acetyl groups [21]. Chitosan has a variety of biological qualities, such as biodegradability, biocompatibility, bacteriostasis, and anti-cancer, lipid-lowering and immunity-enhancing qualities [22]. It is widely used in food additives, for the detection of bioactive molecules, in environmental analysis, in biosensor fields, in biomedical fields and in other everyday chemical industries [23]. The existence of a large number of polar groups, such as the hydroxyl and amino groups, allows chitosan to react with other components [24]. The combination of these two substances will cause changes in the physical and chemical properties, such as adsorption, film-forming, permeability and fiber forming [25,26]. Deep eutectic solvents (DESs) are composed of a hydrogen bond acceptor (HBA) and hydrogen bond donor (HBD) with much lower melting points than those of the individual components [27,28]. DESs have been successfully applied as solvents, additives, templates and monomers in molecular imprinting, and they show excellent properties in the separation of bioactive compounds [29,30,31]. Moreover, when DESs are used on imprinted materials, they can increase the adsorption capacity and the target molecules existing in the aqueous biological matrix [32,33,34]. 

This study aimed to establish a membrane separation method for chlorobenzene compounds from environmental water based on a magnetic molecularly imprinted chitosan membrane (MMICM), and the optimal extraction conditions were optimized. Scanning electron microscopy (SEM), Brunauer–Emmet–Teller (BET) analysis, Fourier transform infrared spectroscopy (FT-IR), thermogravimetric analysis (TGA), and an X-ray diffractometer (XRD) were used to characterize the MMICM. The static adsorption and dynamic adsorption of the material were characterized, which confirmed that the material shows promise for application in the highly selective separation of chlorobenzene compounds.

## 2. Experimental

### Chemicals

Chlorobenzene, 1,2-dichlorobenzene (1,2-DCB), 1,4-dichlorobenzene (1,4-DCB), 1,3-dichlorobenzene (1,3-DCB), 1,2,3-trichlorobenzene (1,2,3-TCB), 1,2,4-trichlorobenzene (1,2,4-TCB), 1,3,5-trichlorobenzene (1,3,5-TCB), 1,2,3,4-tetrachlorobenzene (1,2,3,4-TeCB) 1,2,4,5-tetrachlorobenzene (1,2,4,5-TeCB), choline chloride (ChCl), urea, glycerol, ethylene glycol and acetic acid were purchased from Sigma-Aldrich. Co, Ltd. (Spruce, St. Louis, MO, USA). Chitosan (Mw: 75 kDa, DD: 80 mol%) was obtained from Seafresh Chitosan (Lab) Co. Ltd., Chumphon, Thailand. Acetonitrile, acetone, methanol, ethyl acetate, span-80, glutaraldehyde, isopropanol, liquid paraffin, FeCl_2_·4H_2_O, FeCl_3_·6H_2_O, NH_3_·H_2_O, tetraethyl-orthosilicate (TEOS), methyl methacrylate (MMA), 2,2′-Azobisisobutyronitrile (AIBN) and ethyleneglycol dimethacrylate (EGDMA) were acquired from Chemicals & Metals Co., Ltd. (Jinan, China). 

## 3. Materials Preparation

### 3.1. Synthesis of the DESs

The DESs were prepared by mixing. A ratio of HBAs with HBDs was mixed, stirred and heated under 70 °C for 6 h, and homogeneous colorless DESs were formed. The DESs were as follows: DES-1 (ChCl: urea (1:1, *n/n*)); DES-2 (ChCl: glycerol (1:2, *n/n*)); DES-3 (ChCl: ethylene glycol (1:1, *n/n*)); DES-4 (ChCl: acetic acid (1:2, *n/n*)). It should be stated that only DES-1, at the ratio listed above, was stable and used in the material synthesis process. 

### 3.2. Preparation of the MMICM and MNICM

A total of 500 mg of Fe_3_O_4_ was mixed in 200 mL of the solution (water-ethanol (4:1, *v/v*)) and placed in an ultrasonic bath for 40 min, followed by 10 mL of NH_3_·H_2_O and 4 mL of TEOS, then stirring at room temperature for 12 h. Finally, the resultant product (Fe_3_O_4_-based silica) was collected and dried in the vacuum.

**Figure 1:**
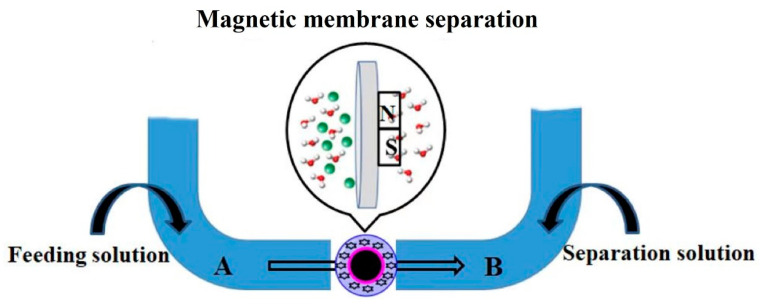
Schematic diagram of the magnetic membrane separation procedure.

First, 0.4 mmol of the chlorobenzene compounds (template), 2.0 mL of the DESs, and 2.0 mmol of MAA were mixed in the solution of 20 mL chloroform and placed in an ultrasonic bath for 1 h. Then, 5.0 g chitosan, 5.0 g Fe_3_O_4_-based silica particles, 0.6 mmol of EGDMA, and 2.4 mmol AIBN were dissolved in 30.0 mL of acetic acid (5.0%) solution and stirred constantly with 20.0 mL of isopropanol. A total of 25 mL paraffin as the dispersing phase and 0.5 mL span-80 as the surfactant agent were then added at 60 °C and stirred (250 rpm) for 60 min. The obtained materials were washed with petroleum ether three times and dried at 60 °C. The synthesized material was pressed into a membrane, named the MMICM, for separation and analysis, and then used in the magnetic membrane separation (MMS) procedure (Figure 1). The magnetic non-imprinted chitosan membrane (MNICM) was obtained in the same way, except without the templates of MIMCM. 

### 3.3. Characterization

The surface morphology was examined by field emission scanning electron microscopy (FE-SEM, SE-4300, MERLIN Compact, ZEISS, Oberkochen, Germany). The FT-IR (IR-Affinity-1, Shimadzu, Kyoto, Japan) spectroscopy was conducted using KBr pellet samples with the wavelength range of 4000 to 400 cm^−1^. The BET surface area of the materials was detected using a pore size analyzer (Gold APP instrument Co., Ltd., Cixi, China) and a V-Sorb 2800TP specific surface area analyzer. The TGA was confirmed by TG 209 (Netzsch) under a nitrogen atmosphere ranging from 25 °C to 800 °C (heating rate: 10 °C·min^−1^). The crystallographic feature was confirmed by the APD 2000 X-ray diffractometer (Italstructures, Riva del Garda, Italy).

### 3.4. Static and Dynamic Adsorption 

A total of 20 mg of MMICM and MNICM was mixed with 1 mL chlorobenzene standard solution at different concentrations (5, 10, 20, 50, 100, 150 and 200 μg·mL^−1^) in a 10 mL beaker, respectively. After mechanical shaking for 90 min at room temperature, the suspension was separated with a magnet and determined by HPLC, and calculated with Equation (1):

(1) $$Q_{e}=\frac{\left(C_{0}-C_{1}\right)V}{m}$$

where V is the volume of the solution; m is the weight of magnetic particles; C_0_ is the concentration of the target molecule (μg·mL^−1^) before adsorption; and C_1_ is the concentration of the target molecule (μg·mL^−1^) after adsorption. 

Then, 20 mg of MMICM or MNICM was dispersed in 1 mL chlorobenzene standard solution (100 μg·mL^−1^) and underwent shaking at room temperature for the dynamic adsorption test. The samples were drawn at different time periods: 1, 2, 5, 10, 15, 30, 60, 90, 120 and 150 min, respectively. The adsorption capacity was calculated with Equation (2):

(2) $$\ln\left(Q_{e}-Q_{t}\right)=\ln(Q_{e})-k_{1}t$$

where k_1_ (min^−1^) is the rate constant of the adsorption; Q_t_ (mg·g^−1^) is the amount adsorbed at the given time; and Q_e_ (mg·g^−1^) is the amount adsorbed at the equilibrium.

### 3.5. Selectivity and Reusability Experiment

To evaluate the adsorption selectivity of MMICM for chlorobenzene, eight structural analogues, including 1,2-DCB, 1,4-DCB, 1,3-DCB, 1,2,3-TCB, 1,2,4-TCB, 1,3,5-TCB, 1,2,3,4-TeCB and 1,2,4,5-TeCB were investigated. A total of 20 mg MMICM and MNICM was added into 2 mL (100 μg·mL^−1^) chlorobenzene and its analogues, respectively, at room temperature. After shaking for 30 min, the adsorption capacity was calculated after injection into the HPLC. The imprinting factor (IF) and selectivity coefficient (SC) were calculated with the Equations:

(3) $$\mathrm{IF}=\frac{Q_{\mathrm{MMICM}}}{Q_{\mathrm{MNICM}}}$$

(4) $$\mathrm{SC}=\frac{{\mathrm{IF}}_{\mathrm{target}}}{{\mathrm{IF}}_{\mathrm{analog}}}$$

where Q_MMICM_ is the adsorption capacity (mg·g^−1^) of MMICM; Q_MNICM_ is the adsorption capacity (mg·g^−1^) of MNICM; IF_target_ is the imprinting factor for the template molecule; and IF_analog_ is the imprinting factor for the analogs.

Rebinding-regeneration cycles were tested to evaluate the stability of the MMICM and MNICM. A total of 20 mg of MMICM or MNICM was dispersed in 2 mL of chlorobenzene standard solution and oscillated for 30 min at room temperature, and the adsorption capacities were analyzed. 

### 3.6. MMS Method

A total of 20 mg of MMICM and MNICM were added to 2 mL of the sample solution, respectively, followed by shaking for 30 min at room temperature. Then, the MMICM and MNICM were separated using an extrinsic magnet, washed with 2 mL of acetonitrile, and eluted using 2 mL of methanol-ammonia (90:10, *v/v*). Finally, the residue was analyzed by HPLC.

### 3.7. Optimization of the MMS Conditions

There are many factors related to the recovery of the MMICM on the chlorobenzene compounds during the MMS procedure. At first, the ratios of the adsorbent and sample volume, the extraction time, and the extraction temperature were investigated. 

It is necessary to optimize the washing and elution conditions to increase the sensitivity and selectivity because of the complexity of the environmental water. For this purpose, different kinds of washing solvents were tested, including methanol, ethanol, acetone, acetonitrile and ethyl acetate solution in the volume range of 0.5 mL to 4.0 mL. Several kinds of eluent solvents (methanol, methanol-5% acetic acid, methanol-5% acetonitrile, methanol-5% ammonium hydroxide and methanol-5% acetone) were tested. 

## 4. Results and Discussion

### 4.1. Static and Dynamic Adsorption 

The adsorption isotherms of MMICM and MNICM for chlorobenzene are shown in Figure S1. In Figure S1a, it can be seen that, for each concentration of chlorobenzene, the adsorption capacity of MMICM was much higher than that of MNICM. 

The kinetic adsorption of the chlorobenzene on the MMICM and MNICM was investigated, with the adsorption time ranging from 1 to 120 min (Figure S1b). It was shown that, within 30 min, there was a clear increase in the amount of the adsorption of chlorobenzene by MMICM and MNICM, and, later, the adsorption efficiency remained basically stable as the time increased. The equilibrium adsorption capacity of MMICM was 102.5 mg·g^−1^, approximately 4.98 times greater than that of MNICM (20.6 mg·g^−1^), indicating that the molecular imprinting technology of MMICM can effectively improve the adsorption performance of adsorbents. 

### 4.2. Characterization

**Figure 2:**
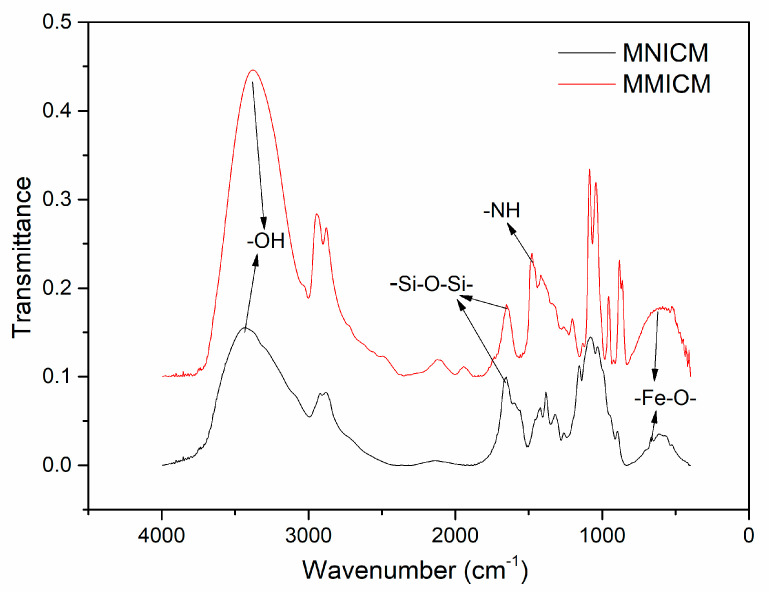
FT-IR spectra of the MNICM and MMICM.

The FT-IR spectra of the MMICM and MNICM are shown in Figure 2. The absorption peak of 580 cm^−1^ was attributed to the Fe-O stretching vibration, indicating that Fe_3_O_4_ was successfully embedded. The absorption peaks at about 1093 cm^−1^, attributed to the Si-O-Si group, and peaks at 1645 and 3419 cm^−1^, attributed to the -OH group, indicated the coating of Fe_3_O_4_ and chitosan. As can be seen, a characteristic feature of the MMICM was the N-H bond at 1550 cm^−1^ of the DESs, in comparison with the MNICM. 

**Figure 3:**
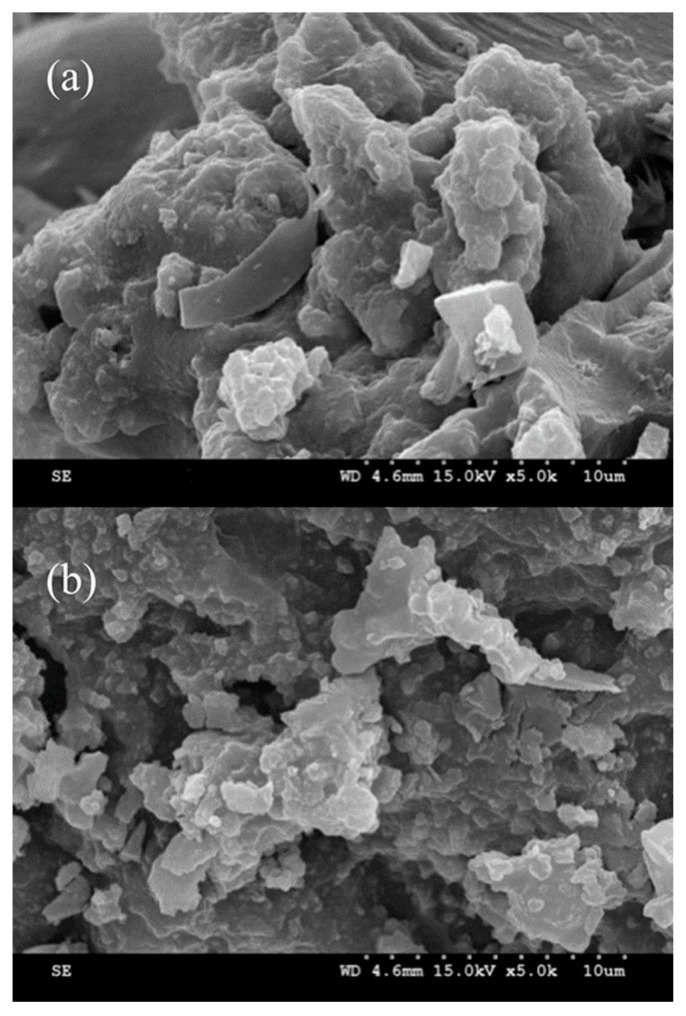
SEM images of the MNICM (**a**) and MMICM (**b**).

The morphologies and microstructures of the constructed MMICM and MNICM are clearly visualized by SEM in Figure 3. As presented here, the SEM micrographs of the MMICM (Figure 3b) showed a more particle-like structure, increasing the adsorption area and performance, compared with the MNICM (Figure 3a). These results demonstrated that the structure of MMICM is well maintained, showing uniform particle size and good dispersion. 

TGA characterization of the MNICM and MMICM was also carried out, as shown in Figure S2. As can be seen, a first mass loss event was tested in the MMICM below 200 °C, and in the MNICM at temperatures below 100 °C, which corresponds to the evaporation of moisture. Then, MNICM started to lose weight significantly at around 100 °C, and stabilized at 18.2% at 400 °C. The temperature at which MMICM began to lose weight increased slightly at about 200 °C, and finally stabilized at 34.8% at 500 °C, indicating the improved thermal stability and increased imprinting groups of the MMICM. The BET from the N_2_ adsorption–desorption assay and the mathematical treatments was used to determine the specific surface areas, pore size distribution, and the volume of MMICM and MNICM, as can be seen in Figure S3. It can be observed that, because of the N_2_ adsorption on the larger mesopores, the adsorption isotherms of both materials increased rapidly at relative pressures higher than 0.9. The surface area of MMICM was 27.0427 m^2^·g^−1^, and that of MNICM was 2.6564 m^2^·g^−1^. These results verified the presence of a mesoporous structure in MMICM, contributing to the great recovery of the analytes.

**Figure 4:**
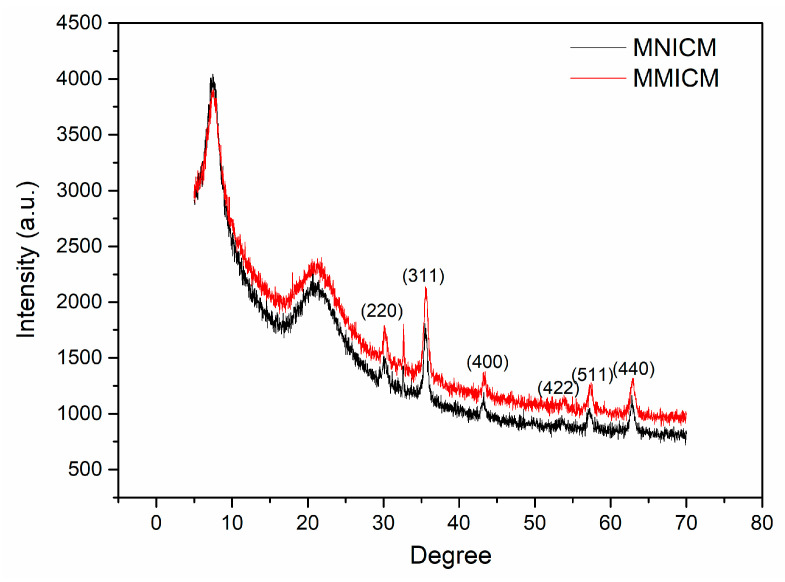
XRD patterns of the MNICM and MMICM.

The XRD patterns for the MMICM and MNICM are shown in Figure 4. In the 2θ range of 5–70°, six characteristic peaks for Fe_3_O_4_ (2θ = 30.32°, 35.58°, 43.14°, 53.45°, 57.10° and 62.66°) were observed, and the peak positions at the corresponding 2θ value were indexed as (220), (311), (400), (422), (511) and (440), respectively, indicating the stability of the magnetic structure, and the presence of the specific diffraction peaks of the synthesized materials.

### 4.3. Optimization Conditions 

#### Effect of the Ratio of Adsorbent and Sample Volumes 

At low concentrations, it is important to study the effect of the ratio of the adsorbent and sample volumes on the extraction ability. Various ratios (3:1, 2:1, 1:1, 1:2, 1:3, 1:4 and 1:5) were examined. In Figure S4, it was found that the recoveries of chlorobenzene obviously decreased with the ratio of 1:2, indicating the higher extraction capability of MMICM, with the higher ratio of the sorbent amount to the sample volume.

In general, the extraction quantity increased with time until an equilibrium was reached. Here, the extraction time was investigated within the range from 5 to 50 min. It was found that 20 min was enough time to elute the chlorobenzene from MMICM, as shown in Figure S5. 

High temperature increases the diffusion rate but has adverse effects on sorption, while low-temperature treatment has positive effects on sorption but has adverse effects on the diffusion rate. The effect of temperature on the extraction efficiency of chlorobenzene from 20 to 50 °C was investigated. Figure S6 shows that the extraction efficiency at 35 °C was the highest, and this was selected for further study.

**Figure 5:**
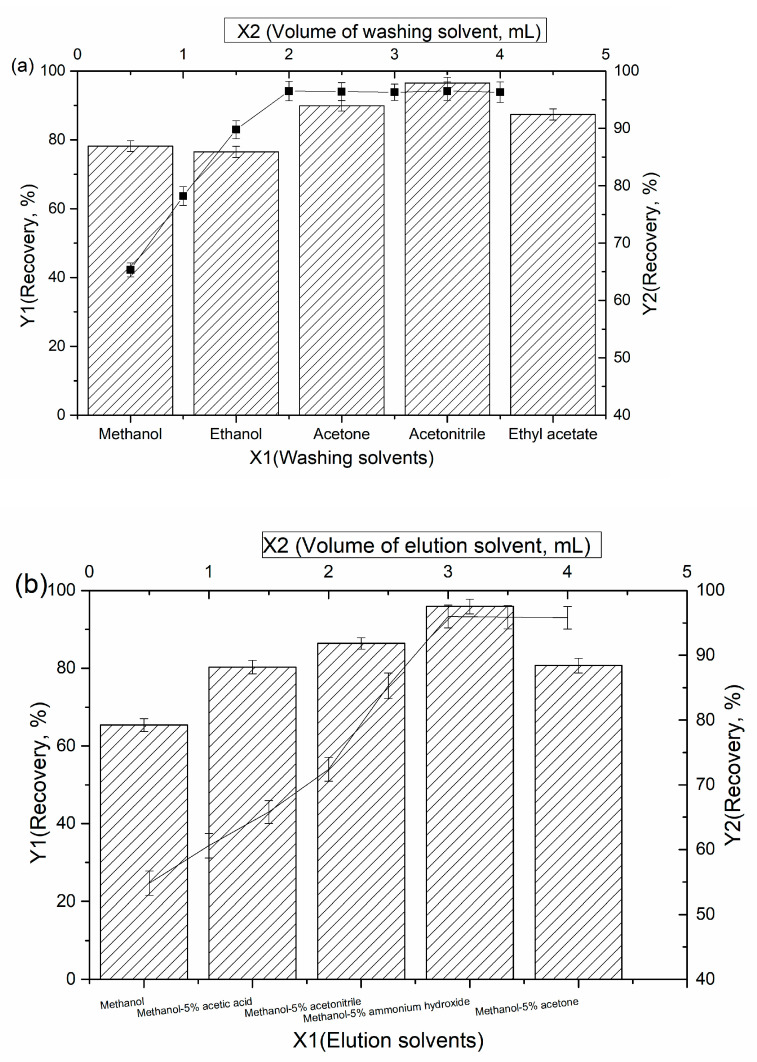
Optimization of the MMS procedure for the chlorobenzene compounds: (**a**) washing solvents, (**b**) elution solvents.

A washing step is necessary to remove other constituents bound to the imprinted sorbent. As shown in Figure 5a, acetonitrile was selected as the washing solvent because of its capability to wash organic compounds from reversed-phase sorbents and its compatibility with the subsequent reaction. A volume range of 0.5–4.0 mL was analyzed to identify the volume of acetonitrile required to wash the chlorobenzene from the cartridge. In this case, 2.0 mL was sufficient to quantitatively wash the other constituents from the adsorbents.

The selection of the appropriate eluting solvent and volume is also very important for the extraction efficiency. As indicated in Figure 5b, the elution of chlorobenzene compounds from the cartridge is performed effectively when using 3.0 mL of methanol-5% ammonium hydroxide as the elution solvent. These relatively low volumes of methanol-5% ammonium hydroxide allow for efficient elution without the further examination with other solvents.

### 4.4. Selectivity and Reusability of MMICM and MNICM

As shown in Figure S7, the MMICM showed obvious higher adsorption capacities for nine compounds compared to MNICM. Moreover, the MMICM exhibited the highest binding capacities for chlorobenzene among all the compounds. The results showed that the MMICM showed a better recognition ability for chlorobenzene, while the MNICM had no specific recognition function. As seen in Table 1, the SC values of the MMICM for chlorobenzene, 1,2-DCB, 1,4-DCB, 1,3-DCB, 1,2,3-TCB, 1,2,4-TCB, 1,3,5-TCB, 1,2,3,4-TeCB and 1,2,4,5-TeCB were 0.22, 0.12, 0.14, 0.13, 0.10, 0.09, 0.10, 0.11 and 0.12, respectively. These results showed that the synthesized MMICM surface successfully generated specific recognition sites for the target molecules.

$$IF=\frac{Q_{\mathrm{MMICM}}}{Q_{\mathrm{MNICM}}},\;\mathrm{SC}=\frac{{\mathrm{IF}}_{\mathrm{target}}}{{\mathrm{IF}}_{\mathrm{analog}}}$$

In order to study the reusability of the imprinted materials, ten cycles of experiments were carried out. As shown in Figure S8, the adsorption rate of MMICM for chlorobenzene was still as high as 94.20% after six cycles, while MNICM was observed with an obvious decrease over the three cycles, indicating that the obtained imprinting material was proven to have good reusability and stability.

### 4.5. Method of Validation and Real Samples Analysis

Five levels of concentrations in the range of 5.00–200.00 μg·mL^−1^ were used to evaluate the accuracy and precision of the method (Table S1), and the precision of the method was determined by the relative standard deviation (RSD), limit of detection (LOD), and limit of quantification (LOQ). The accuracy was obtained in the recovery range 89.02–106.97% for the separation of the chlorobenzene compounds, with the RSD of less than 6.02%. The LOD was in the range of 0.0016–0.057 ng·L^−1^ (3 times) and the LOQ was in the range of 0.0026–0.098 ng·L^−1^ (10 times). The MMICM-MMS was shown to have potential for the separation of chlorobenzene compounds with the proposed method, based on the obtained accuracy and precision data. 

### 4.6. Separation of the Chlorobenzene Compounds from the Environmental Water

**Figure 6:**
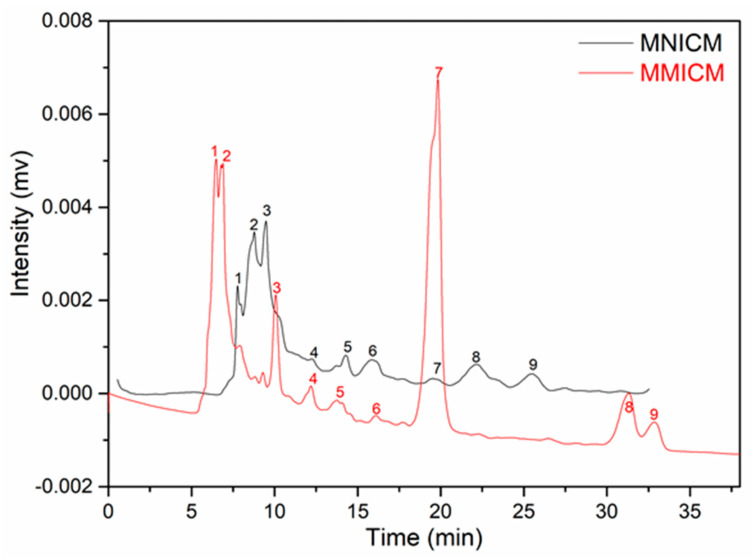
Extraction chromatograms of the environmental water using the MMICM and MNICM. (Column: C18 column, mobile phase: acetonitrile: water (= 70:30, *v*/*v*), flow rate: 1.0 mL·min^−^^1^, UV: 254 nm, injection: 20 μL).

The chromatograms of the water sample after separation using the MMICM and MNICM are displayed in Figure 6 (1:1,2-dichlorobenzene (1,2-DCB), 2:1,4-dichlorobenzene (1,4-DCB), 3:1,3-dichlorobenzene (1,3-DCB), 4:1,2,3-trichlorobenzene (1,2,3-TCB), 5:1,2,4-trichlorobenzene (1,2,4-TCB), 6:1,3,5-trichlorobenzene (1,3,5-TCB), 7:chlorobenzene, 8:1,2,3,4-tetrachlorobenzene (1,2,3,4-TeCB) and 9:1,2,4,5-tetrachlorobenzene (1,2,4,5-TeCB). The result indicated that the MMICM was an excellent material for application in the separation of chlorobenzene compounds from environmental water, and the determination of the chlorobenzene compounds in the water sample was as follows: chlorobenzene (9.64 mg·L^−1^), 1,2-DCB (5.12 mg·L^−1^), 1,4-DCB (5.03 mg·L^−1^), 1,3-DCB (4.93 mg·L^−1^), 1,2,3-TCB (0.98 mg·L^−1^), 1,2,4-TCB (1.16 mg·L^−1^), 1,3,5-TCB (1.21 mg·L^−1^), 1,2,3,4-TeCB (2.18 mg·L^−1^) and 1,2,4,5-TeCB (2.23 mg·L^−1^). 

### 4.7. Comparison with Other Methods

Table 2 shows the comparison between this method and other experimental methods for the separation of chlorobenzene compounds from environmental water. The recoveries of the MMICM-MMS were much higher than those of the other reported methods, and the MMICM-MMS showed higher selectivity for the target analyte separation from the environmental water, owing to the fact that the traditional materials have a poor adsorption performance and low selectivity. Therefore, the developed MMICM-MMS method is an effective and less time-consuming and costly method compared with the traditional separation methods, reducing the interference of the other matrices in the sample and the loss during the separation process.

## 5. Conclusions

The obtained MMICM materials and the MMS method have been successfully developed for the separation of chlorobenzenes in water samples. The favorable extraction effects after the optimization of the extraction conditions (9.64 mg·L^−1^) have been demonstrated, with a 1:2 solid to liquid ratio, a 20 min extraction time and a 35 °C extraction temperature, indicating the greater efficiency, sensitivity, and better extraction efficiency of the method explored here, as compared to the previous methods. 

## Figures and Tables

**Table 1:** Kinds of obtained DESs.

Kinds	MMICM (mg·g^−1^)	MNICM (mg·g^−1^)	IF	SC
Chlorobenze	102.6	20.4	5.03	0.22
1,2-DCB	60.8	19.8	3.07	0.12
1,4-DCB	66.5	19.6	3.39	0.14
1,3-DCB	64.3	19.2	3.35	0.13
1,2,3-TCB	46.5	18.3	2.54	0.10
1,2,4-TCB	42.9	18.2	2.36	0.09
1,3,5-TCB	48.2	18.0	2.68	0.10
1,2,3,4-TeCB	51.6	17.8	2.90	0.11
1,2,4,5-TeCB	53.4	17.4	3.07	0.12

**Table 2:** Comparison of the present method used to separate the chlorobenzene compounds from environmental water with other reported methods.

Materials	Extraction Methods	Detection Methods	Low Limits of Detection	Recovery (%)	Ref.
Meso-/microporous carbon	Solid-phase microextraction	Gas chromatography	(0.003–0.072 ng L^−1^)	90.18–103.02	[35]
Nanoporous silica aerogel	Needle trap extraction	Gas chromatography-mass spectrometry (GC-MS)	0.4–0.8 ng L^−1^	96–101	[36]
Styrene-divinylbenzene	Solid-phase extraction	Gas chromatography	20 ng L^−1^	70–106	[37]
Magnetic molecularly imprinted chitosan membrane	Magnetic membrane separation	High performance liquid chromatography	0.0016–0.098 ng·L^−1^	89.02–106.97	This study

## Data Availability

Not applicable.

## Supplementary Materials

The following supporting information can be downloaded at: https://www.mdpi.com/article/10.3390/polym14153221/s1, Figure S1: Dynamic adsorption (a) and static adsorption (b) of MMICM and MNICM for chlorobenzene; Figure S2: TGA curves of MNICM and MMICM; Figure S3: BET analysis of the MMICM and MNICM; Figure S4: Effect of adsorbent and sample volume on the MMS procedure; Figure S5: Effect of extraction time on the MMS procedure; Figure S6: Effect of extraction temperature on the MMS procedure; Figure S7: Adsorption amounts of different kinds of chlorobenzene compounds by MNICM and MMICM; Figure S8: Reproducibility and stability of MNICM and MMICM. Table S1: Intra-day and Inter-day precisions and accuracies of chlorobenzene compounds.
