# Supplementary material for: The Separation of Chlorobenzene Compounds from Environmental Water Using a Magnetic Molecularly Imprinted Chitosan Membrane
Source: Polymers (Basel). 2022 Aug 8;14(15):3221. doi: 10.3390/polym14153221 (PMC9371115; doi:10.3390/polym14153221)
Supplement: Supplementary file 1 [file polymers-14-03221-s001.zip › polymers-1793432-supplementary.pdf]

# Separation of Chlorobenzene Compounds from Environmental Water Using Magnetic Molecularly Imprinted Chitosan Membrane

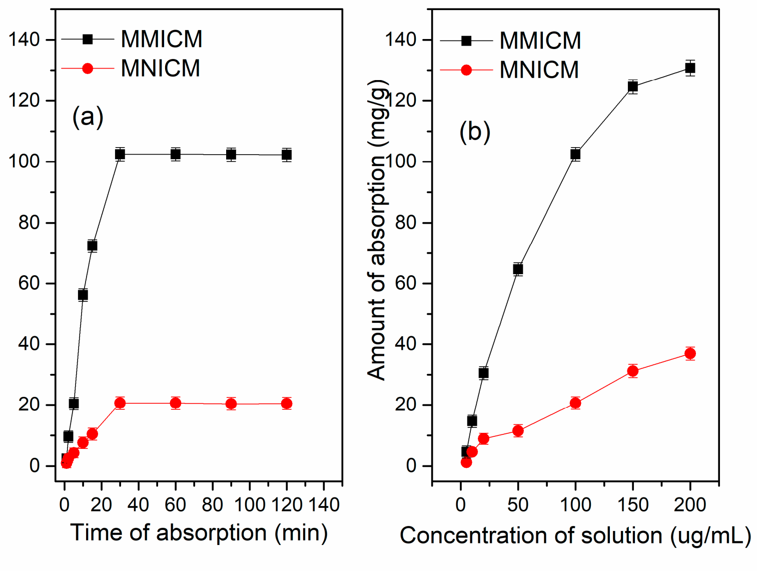


**Figure S1.** Dynamic adsorption (a) and static adsorption (b) of MMICM and MNICM for chlorobenzene.

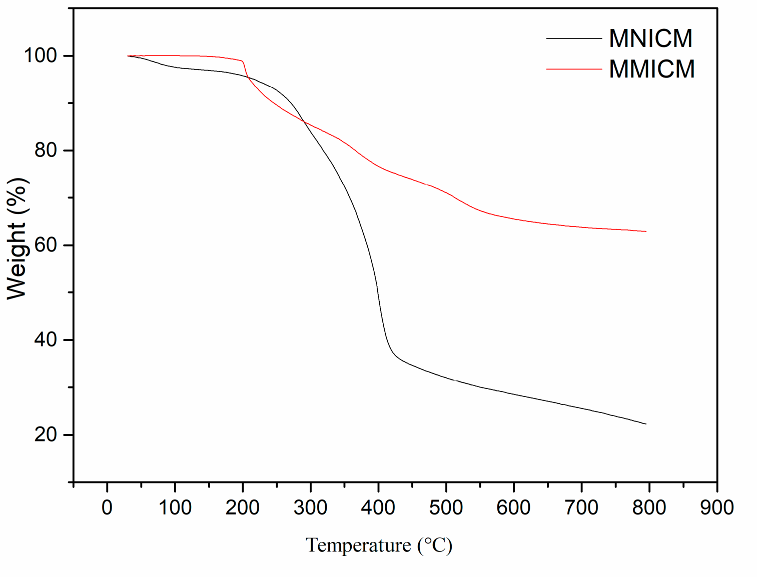


**Figure S2.** TGA curves of MNICM and MMICM.

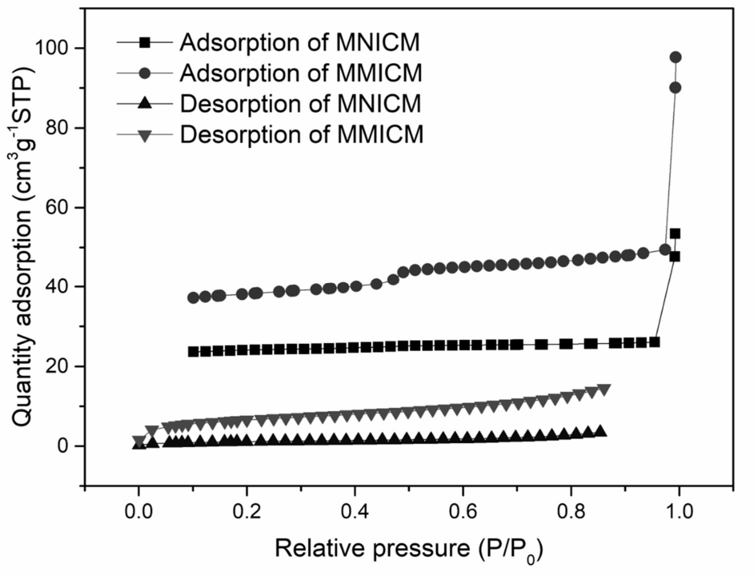


**Figure S3.** BET analysis of the MMICM and MNICM.

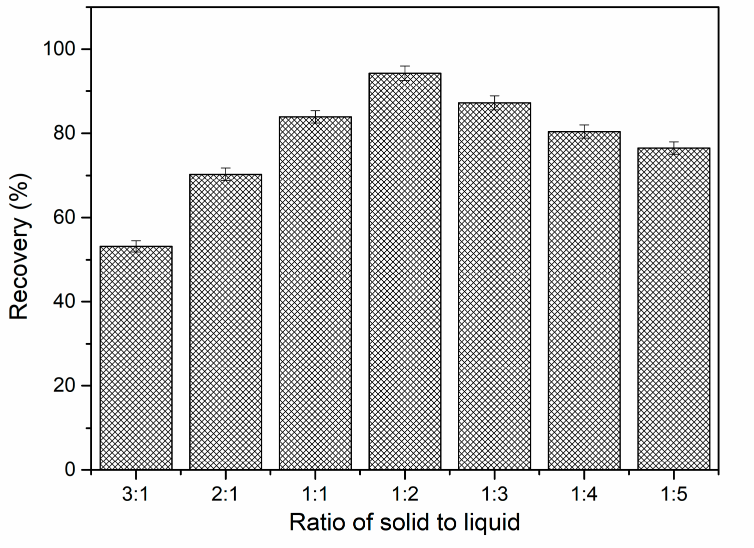


**Figure S4.** Effect of adsorbent and sample volume on the MMS procedure.

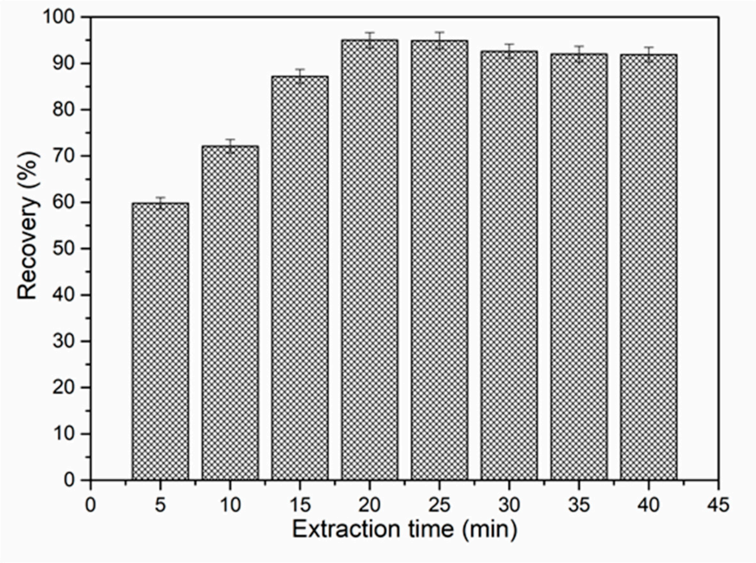

**Figure S5.** Effect of extraction time on the MMS procedure.

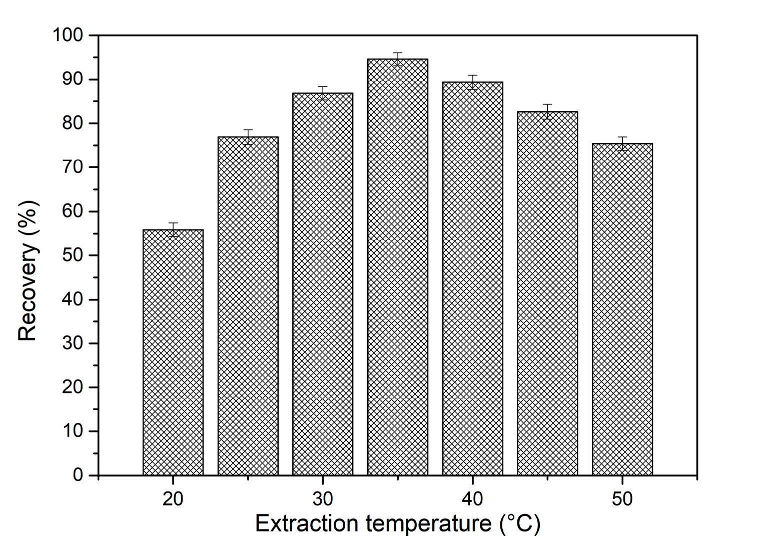


**Figure S6.** Effect of extraction temperature on the MMS procedure.

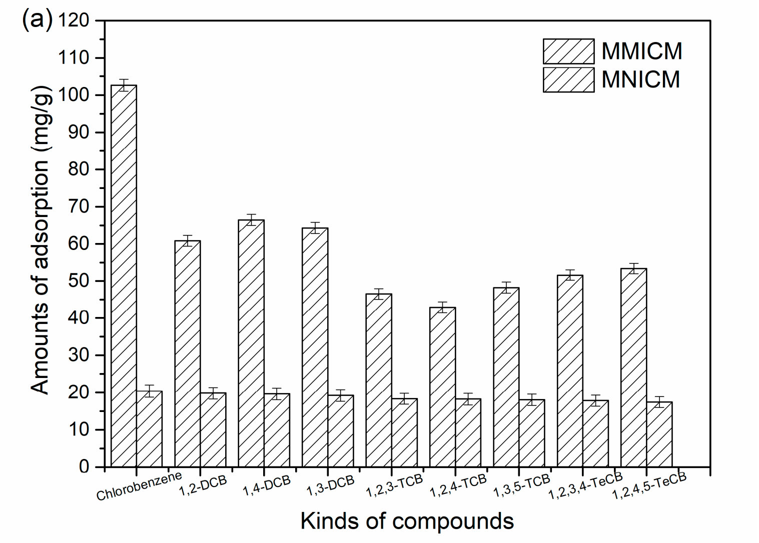


**Figure S7.** Adsorption amounts of different kinds of chlorobenzene compounds by MNICM and MMICM.

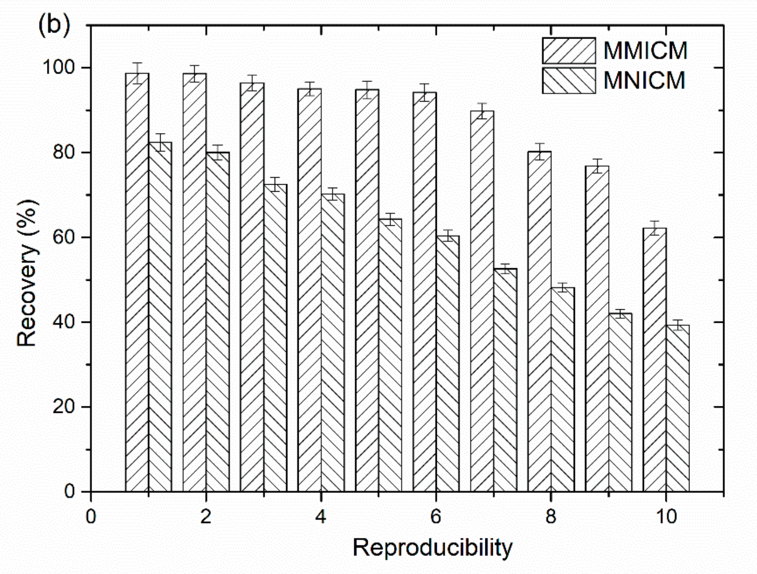


**Figure S8.** Reproducibility and stability of MNICM and MMICM.

Table S1 Intra-day and Inter-day precisions and accuracies of chlorobenzene compounds.

| Targets | Concentration (μg·mL$^{-1}$) | Intra-day | | | | Inter-day | |
|---|---|---|---|---|---|---|---|
| | | LOD (μg·mL$^{-1}$) | LOQ (μg·mL$^{-1}$) | Recovery (%) | RSD (%, n=4) | Recovery (%) | RSD (%, n=4) |
| **Chlorobenzene** | 100 | 0.27 | 0.32 | 97.08 | 4.72 | 96.43 | 5.15 |
| | 200 | 0.16 | 0.26 | 101.06 | 3.83 | 99.51 | 3.12 |
| 1,2-DCB | 100 | 0.31 | 0.46 | 96.89 | 5.12 | 97.13 | 6.02 |
| | 200 | 0.20 | 0.35 | 101.13 | 4.06 | 96.98 | 4.16 |
| 1,4-DCB | 100 | 0.33 | 0.56 | 98.17 | 4.29 | 97.01 | 5.91 |
| | 200 | 0.29 | 0.35 | 99.87 | 3.87 | 98.16 | 3.28 |
| 1,3-DCB | 100 | 0.45 | 0.79 | 106.97 | 5.03 | 96.00 | 4.69 |
| | 200 | 0.39 | 0.68 | 102.52 | 4.01 | 97.13 | 3.12 |
| 1,2,3-TCB | 100 | 0.42 | 0.86 | 95.21 | 4.15 | 92.98 | 5.12 |
| | 200 | 0.36 | 0.57 | 97.00 | 3.21 | 95.07 | 4.65 |
| 1,2,4-TCB | 100 | 0.35 | 0.68 | 95.15 | 4.31 | 92.78 | 5.42 |
| | 200 | 0.27 | 0.45 | 103.54 | 3.01 | 99.12 | 3.75 |
| 1,3,5-TCB | 100 | 0.56 | 0.98 | 93.18 | 4.31 | 92.03 | 5.12 |
| | 200 | 0.48 | 0.67 | 95.02 | 3.08 | 94.13 | 3.46 |
| 1,2,3,4-TeCB | 100 | 0.57 | 0.96 | 90.96 | 4.53 | 89.02 | 4.18 |
| | 200 | 0.45 | 0.82 | 102.17 | 3.96 | 90.13 | 3.24 |
| 1,2,4,5-TeCB | 100 | 0.50 | 0.87 | 95.89 | 4.34 | 91.24 | 4.79 |
| | 200 | 0.39 | 0.65 | 99.12 | 3.03 | 93.42 | 3.83 |

1,2-dichlorobenzene (1,2-DCB), 1,4-dichlorobenzene (1,4-DCB), 1,3-dichlorobenzene (1,3-DCB), 1,2,3-trichlorobenzene (1,2,3-TCB), 1,2,4-trichlorobenzene (1,2,4-TCB), 1,3,5-trichlorobenzene (1,3,5-TCB), 1,2,3,4-tetrachlorobenzene (1,2,3,4-TeCB) and 1,2,4,5-tetrachlorobenzene (1,2,4,5-TeCB)
